# Small field electron beam dosimetry using MOSFET detector

**DOI:** 10.1120/jacmp.v12i1.3267

**Published:** 2010-10-04

**Authors:** Md Nurul Amin, Robert Heaton, Bern Norrlinger, Mohammad K. Islam

**Affiliations:** ^1^ Department of Radiation Physics Princess Margaret Hospital, University Health Network Toronto Ontario Canada; ^2^ Department of Radiation Oncology University of Toronto Toronto Canada

**Keywords:** MOSFET, small field dosimetry, electron beams

## Abstract

The dosimetry of very small electron fields can be challenging due to relative shifts in percent depth‐dose curves, including the location of $d_{\text{max}}$, and lack of lateral electronic equilibrium in an ion chamber when placed in the beam. Conventionally a small parallel plate chamber or film is utilized to perform small field electron beam dosimetry. Since modern radiotherapy departments are becoming filmless in favor of electronic imaging, an alternate and readily available clinical dosimeter needs to be explored. We have studied the performance of MOSFET as a relative dosimeter in small field electron beams. The reproducibility, linearity and sensitivity of a high‐sensitivity microMOSFET were investigated for clinical electron beams. In addition, the percent depth doses, output factors and profiles have been measured in a water tank with MOSFET and compared with those measured by an ion chamber for a range of field sizes from 1 cm diameter to 10 cm× 10 cm for 6, 12, 16 and 20 MeV beams. Similar comparative measurements were also performed with MOSFET and films in solid water phantom. The MOSFET sensitivity was found to be practically constant over the range of field sizes investigated. The dose response was found to be linear and reproducible (within ±1% for 100 cGy). An excellent agreement was observed among the central axis depth dose curves measured using MOSFET, film and ion chamber. The output factors measured with MOSFET for small fields agreed to within 3% with those measured by film dosimetry. Overall results indicate that MOSFET can be utilized to perform dosimetry for small field electron beam.

PACS number: 87.55.Qr

## I. INTRODUCTION

Small superficial cancerous lesions are typically treated with electrons. Depending upon the size, shape and location of the lesion, the field aperture, commonly referred to as cutout, is custom‐made using cerrobend. Accurate dosimetry of such small electron fields usually requires dosimetric measurement. Accurate dosimetry for such small fields is challenging due to the loss of lateral electronic equilibrium within the field. This can result in a shift of $d_{\text{max}}$ towards the surface and other modifications of the depth dose curve, as well as a change in the beam profile characteristics at depth. The use of such fields in the clinic thus requires careful and detailed measurements to characterize both the output and coverage achieved.

Small field dosimetry is typically achieved using film dosimetry.^(^ ^1^ ^)^ However, modern radiotherapy clinics have increasingly reduced their use of film, making it difficult to maintain development systems in a condition to support dosimetric measurements. Many new clinics have eliminated film processing completely from the clinical area so that dosimetric film processing is no longer an option. While radiochromic film can be used for these measurements,^(^ ^2^ ^–^ ^5^ ^)^ this dosimetry system requires specialized equipment which may not be available in some clinics, in addition to a careful measurement procedure to obtain accurate measures. Consequently, other dose measurement methods need to be considered, especially for use in routine clinical measurements which need to be assessed in time periods shorter than a day.

Conventional dosimetry equipment, such as Farmer type ion chambers and standard parallel plate chambers, is difficult to use for small electron field measurements due to the loss of electronic equilibrium and partial volume irradiation effects.^(^ ^6^ ^,^ ^7^ ^)^ Small area detectors such as diodes can be used, but are typically used in water and cannot be easily positioned in the water‐equivalent plastics favored for routine clinical work. In recent years, a new type of solid state dosimeter, the MOSFET detector, has been introduced into the clinic, primarily for *in vivo* patient dosimetry. In this work, we have investigated extending the use of the MOSFET to small field electron dosimetry.

MOSFET dosimeters have been widely studied for photon beam dosimetry^(^ ^8^ ^–^ ^14^ ^)^ and have been used extensively for *in vivo* clinical dose measurements,^(^ ^11^ ^,^ ^12^ ^,^ ^15^ ^–^ ^19^ ^)^ as well as for small field photon dosimetry.^(^ ^20^ ^)^ MOSFET systems have recently been investigated for use in electron beam dosimetry,^(^ ^21^ ^–^ ^23^ ^)^ as well. In this study, the application of the mobileMOSFET dosimeter system (Best Medical Canada, Ottawa, ON) to small field electron dosimetry is characterized and compared to conventional film and ionization chamber systems for determining required critical parameters for patient treatments.

## II. MATERIALS AND METHODS

The mobileMOSFET system (TN‐RD‐70‐W), along with a set of high‐sensitivity microMOSFET (TN‐1002RDM), was used for this investigation. The sensitive detector region has dimensions of $0.2\times 0.2\;{\text{mm}}^{2}$, with a layer thickness of 0.5 μm. In all measurements described here, the detector was oriented with the smallest dimension aligned to the beam axis. MOSFETs were irradiated under high bias setting. Dose measurements were made on a Varian linear accelerator (Clinac 2100EX, Varian Medical Systems, Palo Alto, CA), which provides 6, 9, 12, 16 and 20 MeV electron beams. Small circular fields ranging from 1 to 6 cm in diameter were fabricated, using cerrobend, for the $6\times 6\;{\text{cm}}^{2}$ applicator.

First, the reproducibility, linearity and sensitivity of MOSFET were evaluated for all electron energies using a $10\times 10\;{\text{cm}}^{2}$ field and by placing the MOSFET at the respective $d_{\text{max}}$ depth in the solid water phantom. Subsequently, extensive measurements have been made for small fields using MOSFET, film and ion chamber. Each MOSFET was separately calibrated at a normalization depth of 1.5 cm in solid water for 6 MeV, 2.0 cm for 9 MeV and 3.0 cm for 12, 16 and 20 MeV.

The dose versus optical density calibration curve for the films (Kodak X‐Omat V films, Eastman Kodak Company, Rochester, NY) were determined for each of the electron energies. For point dosimetry, the films were read using a manual densitometer (Macbeth, model:TD932, Kollmorgen Instruments Corporation, New Windsor, NY). The relative dose profiles were read off the films using VXR‐16 DOSIMETRYPRO scanner (Vidar System Corporation, Herndon, VA) and analyzed by RIT113 commercial software (V4.4, Radiological Imaging Technology Inc., Colorado Springs, CO).

The selection of appropriate measuring devices is particularly important for small field electron dosimetry. To illustrate this point, a range of equipment available to a typical radiotherapy clinic was used to perform dosimetry measurements. The relative output in phantom at a depth of the nominal dose maximum ($d_{\text{max}}$ for $10\times 10\;{\text{cm}}^{2}$ field) was determined using MOSFET, film and ion chamber for 50 MU irradiations using various field sizes. A commonly available parallel plate ion chamber (Capintec Inc, Ramsey, NJ: $0.5\;{\text{cm}}^{3}$ active volume, 1.6 cm active volume diameter, 2 cm cavity diameter) was used. Measurements were performed in a $40\times 40\times 15\;{\text{cm}}^{3}$ solid water phantom with 6, 12, 16 and 20 MeV electron beams.

### A. Percentage depth dose (PDD)

**Figure 1:**
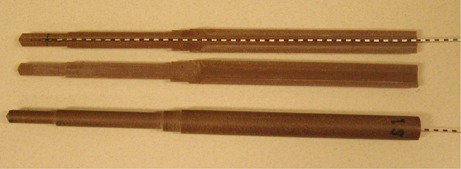
Solid water MOSFET holder to keep MOSFET in the desired position.

To investigate the performance of MOSFET at various depths, the percentage depth doses for 12 MeV electrons using $10\times 10\;{\text{cm}}^{2}$ field were measured and compared with those obtained by an ion chamber (NE2571, Thermo Electron Corporation, Cambridge, UK) in a water tank (Med‐Tec Inc., South Plainfield, NJ). Corresponding measurements were performed with film stacked in between solid water slab and positioned perpendicular to the beam axis. Film depths within the stack were corrected to account for film thickness. To take into account the effective point of measurement of the cylindrical ion chamber, the measured depth ionization data were shifted upstream by 0.5 times the radius of the chamber cavity. A special holder (Fig. 1) was designed to keep the MOSFET in the desired position inside the water tank.

### B. BEAM PROFILE

To assess the performance of MOSFET in the crossbeam dimension of electron beams, profiles of 6, 12, 16 and 20 MeV electrons were measured at 1.5 cm depth in a water tank for 3 cm diameter circular field and compared with those measured with film (in solid water).

### C. OUTPUT FACTOR

The output factor, as defined by the ratio of the dose at $d_{\text{max}}$ for the field of interest to the dose at $d_{\text{max}}$ for the $10\times 10\;{\text{cm}}^{2}$ field were measured for small fields using MOSFET, as well as with films. The points of measurements (i.e., the depths of corresponding maximum dose) were determined from the respective PDD measured in the Med‐Tec water tank using MOSFET.

## III. RESULTS

The MOSFET responses were found to be highly linear ($R^{2}=1$) over the measured dose range of 1 cGy to 1000 cGy. The reproducibility, defined as the standard deviation of 12 sequential measurements expressed as a percent of mean value at the dose level of 100 cGy, was found to be <1%. The dose‐responses for 6, 9, 12, 16 and 20 MeV electrons were found to be 7.28±0.05, 7.34±0.06, 7.28±0.06, 7.34±0.01, and 7.32±0.04 mV/cGy, respectively. These results indicate that the electron energy does not significantly influence the MOSFET sensitivity.

**Figure 2:**
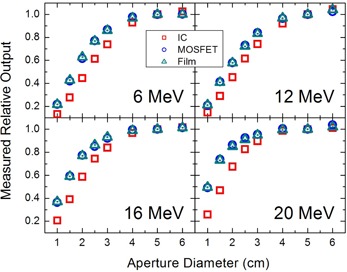
Relative dose factors for circular apertures at nominal $d_{\text{max}}$, normalized to the $10\times 10\;{\text{cm}}^{2}$ field. Measurements were performed for each of 4 electron beam energies using a parallel plate chamber (red □), MOSFET (green Δ) and film (blue O).

The relative dose factors, measured at the nominal $d_{\text{max}}$, are presented in Fig. 2. The results obtained by MOSFET, film and ion chamber agreed well for field sizes equal to and higher than 4 cm diameter. For the smaller field sizes, however, the relative dose factors measured by the ion chamber deviates significantly (up to 47.9%) from those measured by MOSFET and film. These discrepancies are due to the large cavity dimensions (2 cm diameter) of the ion chamber relative to the field width, which consequently perturb the dose measurement.^(^ ^24^ ^–^ ^26^ ^)^ Overall, the results obtained by MOSFET were found to agree with films to within 3%.

**Figure 3:**
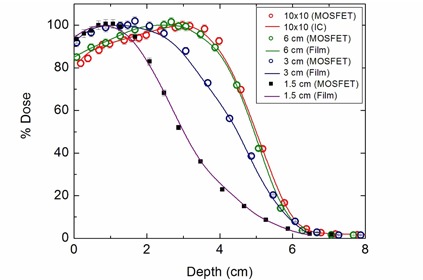
Performance of MOSFET in determining the variation of central axis percentage depth dose (PDD) for 12 MeV electrons, as a function of field size. For $10\times 10\;{\text{cm}}^{2}$ field the reference PDD was measured with ion chamber (model: NE2571, 0.6 cc) and for smaller fields the PDD were measured with film.

Comparative central axis percentage depth dose (PDD) data for 12 MeV beams measured with film, ion chamber and MOSFET are shown in Fig. 3. The figure clearly demonstrates the significant shifts of PDD and $d_{\text{max}}$ with change in field size towards the surface. As shown, the agreements among the data were found to be within 2% (in the low‐gradient region) and within 2 mm (in the high‐gradient region). The summary of the agreements among the interesting depth dose parameters ($d_{\text{max}}$, $d_{90}$, $d_{80}$ and $d_{50}$) are presented in Table 1.

**Table 1:** Measured depth of clinical parameters for 12 MeV beams $d_{\text{max}}$, $d_{90}$, $d_{80}$ and $d_{50}$ for 1, 1.5, 2, 2.5, 3, 4, 6 cm circular fields and $10\times 10\;{\text{cm}}^{2}$ field.

*Cutout Size*		*1.5 cm*	*3 cm*	*6 cm*	$10\times 10\;{\text{cm}}^{2}$
$d_{\text{max}}$ (cm)	Film	0.85	1.6	2.8	3.0
	MOSFET	0.9	1.7	3.0	3.0
$d^{90}$ (cm)	Film	1.7	2.8	3.8	3.9
	MOSFET	1.7	2.8	3.7	3.9
$d^{80}$ (cm)	Film	2.1	3.3	4.25	4.3
	MOSFET	2.1	3.4	4.1	4.3
$d^{50}$ (cm)	Film	3.0	4.5	4.9	5.0
	MOSFET	2.8	4.4	4.85	5.05

**Figure 4:**
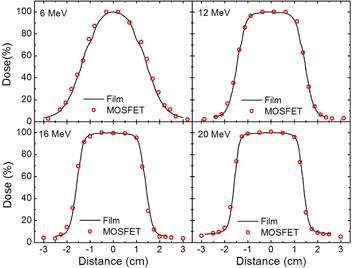
Dose profiles at the depth of 1.5 cm, measured with film (—) and MOSFET (O) for 3 cm circular field.

The 6, 12, 16, and 20 MeV crossbeam profiles are presented in Fig. 4 for the 3 cm circular field measured at 1.5 cm depth utilizing MOSFET and film dosimeters. The MOSFET data agrees within 1 mm with the profile measured by the film.

**Figure 5:**
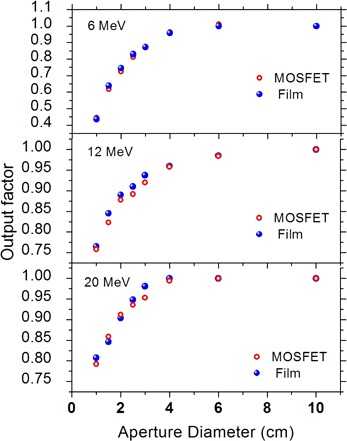
A comparison of output factors measured using MOSFET and film for the circular field sizes of 1, 1.5, 2, 2.5, 3, 4, and 6.

The output factors for small fields measured by MOSFET and film are shown in Fig. 5. The average agreements between the results obtained by MOSFET with those by film were found to be (0.49%±1.86%), (1.13%±0.98%) and (0.53%±1.44%) for 6, 12 and 20 MeV beams, respectively. The maximum deviation was 3.1% for 6 MeV beam with a 1.5 cm diameter cutout.

## IV. DISCUSSION

The results presented in this report clearly demonstrate the challenges associated with the dosimetry of small field electron beams. One must take into account the shifts in the PDD for small fields, with respect to the reference PDD. Otherwise, substantial error may be made in the dose coverage of the target volume. Furthermore, a significant error can be made if an appropriate detector is not used in measuring the relative dose output. The performance of MOSFET with its response linearity, insensitivity to energy variation and small size (being a “point detector”) makes it suitable as a practical dosimeter for small field electron dosimetry.

The work performed for this report required time‐consuming, point‐by‐point measurement with MOSFET for PDD and crossbeam profiles. However, suitable arrays of MOSFET and MOSFET holder can be developed for PDD and output measurements.

## V. CONCLUSIONS

In this work the challenges associated with the dosimetry of small electron beams have been demonstrated. The PDD and $d_{\text{max}}$ shift towards the surface with changes in field size. Measurement of output for field sizes less than 4 cm diameter can be substantially lower if a regular size detector is used. The use of MOSFET detectors has been investigated for various small fields with a range of electron energies. The results indicate that MOSFET detectors can be used for routine clinical dosimetry of electron beams.
